# A comparative study of structural parameters for spiral groove bearing in centrifugal rotary blood pump

**DOI:** 10.1016/j.heliyon.2023.e14313

**Published:** 2023-03-06

**Authors:** Cong Xue, Yu Chen, Bin Zhu, Xiuying Wang

**Affiliations:** a Department of Cardiology, The Third Affiliated Hospital of Soochow University, Changzhou 213003, China; b School of Mechanical Engineering, Jiangsu University of Technology, Changzhou 213001, China; c Department of Critical Care Medicine, The Third Affiliated Hospital of Soochow University, Changzhou 213003, China

**Keywords:** Spiral groove bearing, Structural parameter, Hydrodynamic performance, Blood film

## Abstract

In this work, the hydrodynamic characteristics of spiral groove bearing in centrifugal rotary blood pump is investigated for the cases with different structural parameters. The simulation model is proposed based on the CFD technology and the effectiveness of simulation model is demonstrated by the published data. Then, the pressure, load carrying capacity and friction torque are calculated and the characteristics of pressure distribution are analyzed. It is found that the structural parameters of spiral groove would lead to the complex pressure distribution of blood film and the load carrying capacity also changes at the same time. Moreover, the deep analysis of structural characteristics for spiral groove bearing is conducted based on the orthogonal design method, which could improve the computational efficiency of hydrodynamic behavior of spiral groove bearing. And, the mapping relationship between structural parameter and hydrodynamic performance of bearing preferably is also illustrated.

## 1 Introduction

Centrifugal pumps have an extensive prospect in clinical applications as a new generation of cardiopulmonary bypass (CPB) power device because of small size, light weight, high safety coefficient, large flow rate and minor damage to blood components [1]. At present, centrifugal pumps are widely used in extracorporeal membrane oxygenation (ECMO), left heart assist and conventional CPB heart surgery. It is well known that the spiral groove bearing plays an important role in the centrifugal pumps [2,3]. Then, the structural characteristics of spiral groove bearing can decide the hydrodynamic performance of centrifugal rotary blood pump, which includes pressure, load carrying capacity and energy loss. However, the two difficult technology problems should be solved. The first question is the description of physical characteristics for blood film, which includes the pressure distribution and velocity distribution. The other one is that the mapping relationship between structural parameters and hydrodynamic performance of spiral groove bearing [[4], [5], [6]]. Therefore, it is valuable to establish an available model for illustrating the hydrodynamic behavior of spiral groove bearing and the effects of spiral groove parameters on the hydrodynamic performance should be represented.

The effects of structural characteristics for spiral groove are attracted researchers [[7], [8], [9]]. Han and Zou [10] proposed a new structural feature of spiral groove bearing in centrifugal rotary blood pump. The relationship between structural parameters and hydrodynamic performance of spiral groove bearing were revealed. Ma [11] investigated the hydrodynamic behavior of spiral groove bearing at low pressure and the relationship between rotational speed and opening force were displayed. Considering the effects of inner and turbulence, Lin and Jiang [12] established the hydrodynamic analysis model of spiral groove bearing. The results showed that the inertia force could increase the maximum pressure and friction torque. Hu [13] presented a numerical model for representing the transient lubrication performance of spiral groove bearing, which indicated the variation of groove depth might change the friction torque. Based on the theory and experiment, Sakota [14] investigated the influence of groove structural on the hydrodynamic response of spiral groove bearing and the design of groove structural parameter played an important role in the spiral groove bearing.

It is important to note that many investigations focus on the structural design of spiral groove bearing [15,16]. Fesanghary [17] investigated the effects of radius ratio and aspect ratio on the load carrying capacity of bearing. Based on the optimum groove shapes, the load carrying capacity of bearing was improved 36%. Felipe [18] applied the numerical model for hydrodynamic performance analysis of spiral groove bearing. The change of groove depth improved the flow characteristics of blood. Ochiai [19] applied a comprehensive method for studying the influence of spiral angle characteristics on the hydrodynamic behavior of bearing and the stiffness of bearing was improved by the optimum groove shape. In addition, the numerical study illustrate that the structural parameters effected on the hydrodynamic behavior of blood pump, which was conducted by Liu [20]. The change of structural parameter could cause the variation of flow field near the blood pump bearing. A comparative study of optimizing hydrodynamic levitated centrifugal blood pump was developed by Kosaka and Sakota [21]. In this work, the experiment of bearing was design and the test data validated the effectiveness of hydrodynamic analysis model.

In this work, the hydrodynamic analysis model of spiral groove bearing in centrifugal rotary blood pump is established, which considers the structural characteristics of spiral groove and pressure boundary conditions. Then, the effects of structural parameters on the pressure distribution and load carrying capacity are discussed. Moreover, the orthogonal design approach is employed to investigate the hydrodynamic behavior of spiral groove bearing with different structural characteristics.

## 2 Model of spiral groove bearing

**Fig. 1:**
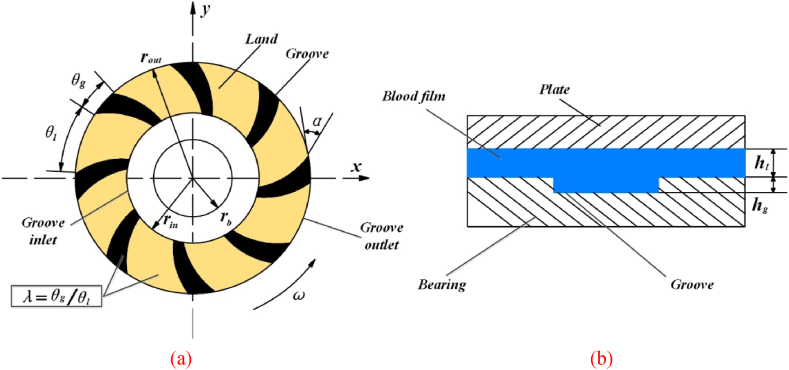
Geometrical configuration of spiral grooved bearing.

The spiral groove bearing of centrifugal rotary blood pump is plotted in Fig. 1. It is known that the structural characteristics of spiral groove plays an important role in the hydrodynamic performance of bearing [22,23]. $r_{in}$ is the inner radius, $r_{out}$ denotes the outer radius and *ω* is the rotational speed of spiral groove bearing. Then, the characteristics of spiral groove can be described by logarithmic spiral, that is,

(1) $$r=r_{b}e^{\theta \;\tan\;\alpha }$$

where $r_{b}$ is the based radius of spiral curve, θ represents the circumferential coordinate in polar coordinates and spiral angle (α) can be displayed.

The thickness of blood film at the land area ($h_{l}$) and groove area ($h_{g}$) can be expressed by:

(2a) $$h=h_{l}$$

(2b) $$h=h_{l}+h_{g}$$

### 2.1 Numerical analysis model

The hydrodynamic behavior of blood film could decide the performance of spiral groove bearing and the description of hydrodynamic characteristics of blood film plays an important role [24,25]. With the assumptions of laminar and isothermal flow, the characteristics of blood film should be satisfied the Navier-Stokers equations, that is,

(3) $$\left\{ \begin{array}{c} \frac{du}{dt}=-\frac{1}{\rho }\frac{\partial p}{\partial x}+\mu \left(\frac{\partial^{2}u}{\partial x^{2}}+\frac{\partial^{2}u}{\partial y^{2}}+\frac{\partial^{2}u}{\partial z^{2}}\right)\\ \frac{dv}{dt}=-\frac{1}{\rho }\frac{\partial p}{\partial y}+\mu \left(\frac{\partial^{2}v}{\partial x^{2}}+\frac{\partial^{2}v}{\partial y^{2}}+\frac{\partial^{2}v}{\partial z^{2}}\right)\\ \frac{dw}{dt}=-\frac{1}{\rho }\frac{\partial p}{\partial z}+\mu \left(\frac{\partial^{2}w}{\partial x^{2}}+\frac{\partial^{2}w}{\partial y^{2}}+\frac{\partial^{2}w}{\partial z^{2}}\right) \end{array}\right.$$

where u, v and w are the velocity components of fluid in the direction of *x*, *y* and *z*, respectively. p is the pressure of blood film and ρ denotes fluid density. μ and t are the kinematic viscosity and time.

In addition, the mass conservation equation is employed in the model of blood film, which is expressed as:

(4) $$\frac{\partial \rho }{\partial t}+\frac{\partial \left(\rho u\right)}{\partial x}+\frac{\partial \left(\rho v\right)}{\partial y}+\frac{\partial \left(\rho w\right)}{\partial z}=0$$

Meanwhile, the momentum conservation equations are also applied in the hydrodynamic behavior of spiral groove bearing, that is,

(5) $$\left\{ \begin{array}{c} \frac{du}{dt}=\rho f_{x}-\frac{\partial p}{\partial x}+\frac{\partial }{\partial z}\left[\mu \left(\frac{\partial w}{\partial x}+\frac{\partial u}{\partial z}\right)\right]+\frac{\partial }{\partial x}\left[2\mu \frac{\partial u}{\partial x}+\lambda \left(\frac{\partial u}{\partial x}+\frac{\partial v}{\partial y}+\frac{\partial w}{\partial z}\right)\right]+\frac{\partial }{\partial y}\left[\mu \left(\frac{\partial u}{\partial y}+\frac{\partial v}{\partial x}\right)\right]\\ \frac{dv}{dt}=\rho f_{y}-\frac{\partial p}{\partial y}+\frac{\partial }{\partial x}\left[\mu \left(\frac{\partial u}{\partial y}+\frac{\partial v}{\partial x}\right)\right]+\frac{\partial }{\partial y}\left[2\mu \frac{\partial v}{\partial y}+\lambda \left(\frac{\partial u}{\partial x}+\frac{\partial v}{\partial y}+\frac{\partial w}{\partial z}\right)\right]+\frac{\partial }{\partial z}\left[\mu \left(\frac{\partial v}{\partial z}+\frac{\partial w}{\partial y}\right)\right]\\ \frac{dw}{dt}=\rho f_{z}-\frac{\partial p}{\partial z}+\frac{\partial }{\partial y}\left[\mu \left(\frac{\partial v}{\partial z}+\frac{\partial w}{\partial y}\right)\right]+\frac{\partial }{\partial z}\left[2\mu \frac{\partial w}{\partial z}+\lambda \left(\frac{\partial u}{\partial x}+\frac{\partial v}{\partial y}+\frac{\partial w}{\partial z}\right)\right]+\frac{\partial }{\partial x}\left[\mu \left(\frac{\partial w}{\partial x}+\frac{\partial u}{\partial z}\right)\right] \end{array}\right.$$

where f is the external body forces and λ represents the second molecular viscosity.

The load carrying capacity could describe the performance of spiral groove bearing, which is obtained by the pressure distribution of blood film [26,27]. Then, it is necessary to give the expression of load carrying capacity of spiral groove bearing, which is written as:

(6) $$W=\int_{0}^{2\pi }\int_{R_{1}}^{R_{2}}prdrd\theta$$

where *p* denotes the blood film pressure.

And, the friction can be given by:

(7) $$F=-\int_{0}^{2\pi }\int_{R_{1}}^{R_{2}}\tau drd\theta$$

where τ is the shear force of blood film surface.

### 2.2 Orthogonal design approach

In order to improve the accuracy of hydrodynamic analysis for spiral groove bearing, the structural parameters of groove are always chosen detailedly. The more parameters would increase the calculation duration and complexity of analysis. Then, the design approach of spiral groove bearing is established by the orthogonal experiment method. For example, the number of structural parameters is *m* and the each structural parameter contains *r* values. If all cases of structural parameters can be conducted, the number of cases is *m*^r^ times [28,29]. As the increase of structural parameters, the number of case would growth and the hydrodynamic performance analysis of spiral groove bearing becomes more difficulty. Based on orthogonal design approach, the orthogonal table ($L_{n}\left(r^{m}\right)$) of structural parameters can be established, which considers the repeatability of structural parameters and variables. Then, the workload of computation and analysis would be reduced obviously. Moreover, the definition of average value ($k_{i}$) and extreme value (*R*) can provide helpful for the analysis of structural parameters for spiral groove bearing, which is given as:

(8) $$k_{i}=\sum A_{i}\;\left(i=1,2,\cdots ,r\right)$$

(9) $$R=\max\left\{k_{1},k_{2},\cdots ,k_{r}\right\}-\min\left\{k_{1},k_{2},\cdots ,k_{r}\right\}$$

**Fig. 2:**
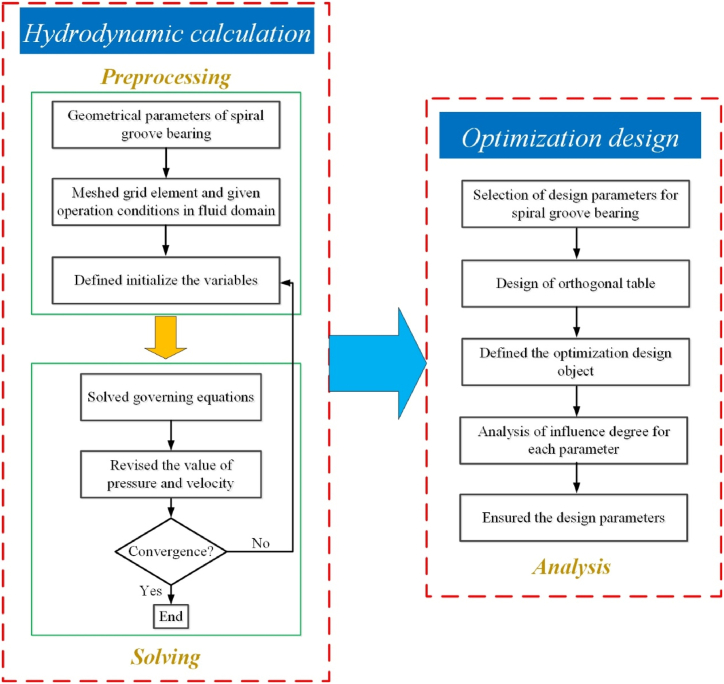
The numerical method flowchart of spiral groove bearing.

In addition, the numerical method flowchart of spiral groove bearing is displayed in Fig. 2. In the hydrodynamic characteristics calculation section, the geometrical parameters of spiral groove bearing should be defined, and the model of spiral groove bearing is divided to grid element. And then, the operation conditions in fluid domain is given out. Moreover, the variables is initialized, and the hydrodynamic characteristics (pressure, velocity, etc) are calculated based on the governing equations. During the solution, the values of pressure and velocity are constantly revised. If the residual curve is convergence, the calculation is completed. According to the design parameters of spiral groove bearing, the orthogonal table is established and the optimization design object is listed. Based on the analysis of influence degree for each parameter, the structural parameters of spiral groove bearing can be ensured.

## 3 Case study

**Fig. 3:**
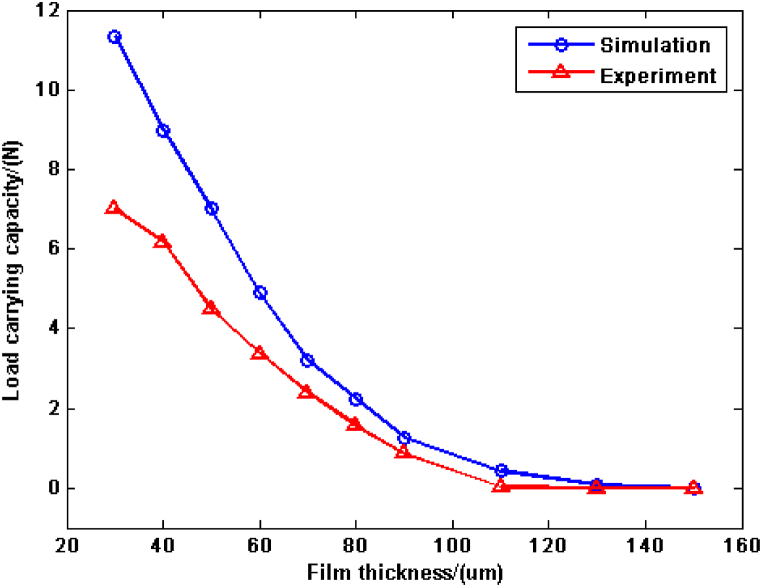
The load carrying capacity of spiral groove bearing with different film thicknesses.

In order to demonstrate the effectiveness of the presented method, the simulation results are compared with the published data in Ref. [17]. The structural parameters of spiral groove bearing includes inner radius (7 mm), outer radius (19 mm), based radius (5 mm), groove angle (12°), groove width ratio (0.75), groove depth (0.169 mm) and groove number (10). Then, the rotational speed is chosen 2500 rpm. In the CFD model, the SIMPLE algorithm and pressure-velocity coupling are employed and the maximum convergence residual is defined as 10^−5^. The simulation results and experiment data are listed in Fig. 3. As the growth of film thickness, the load carrying capacity of spiral groove bearing decreases. The variation trend of load carrying capacity by the presented method is similar with the experiment data in Ref. [17]. The reason of this phenomenon can be concluded that the increase of film thickness would decrease the compress of blood film molecule. And then, the load carrying capacity decreases in the same condition, especially in the little film thickness. Although the difference of simulation results and experiment data appears, the maximum deviation is only 4.34 N and it is acceptable in the engineering calculation, which illustrates the proposed method in this work could show the hydrodynamic behavior of spiral groove bearing adequately.

**Fig. 4:**
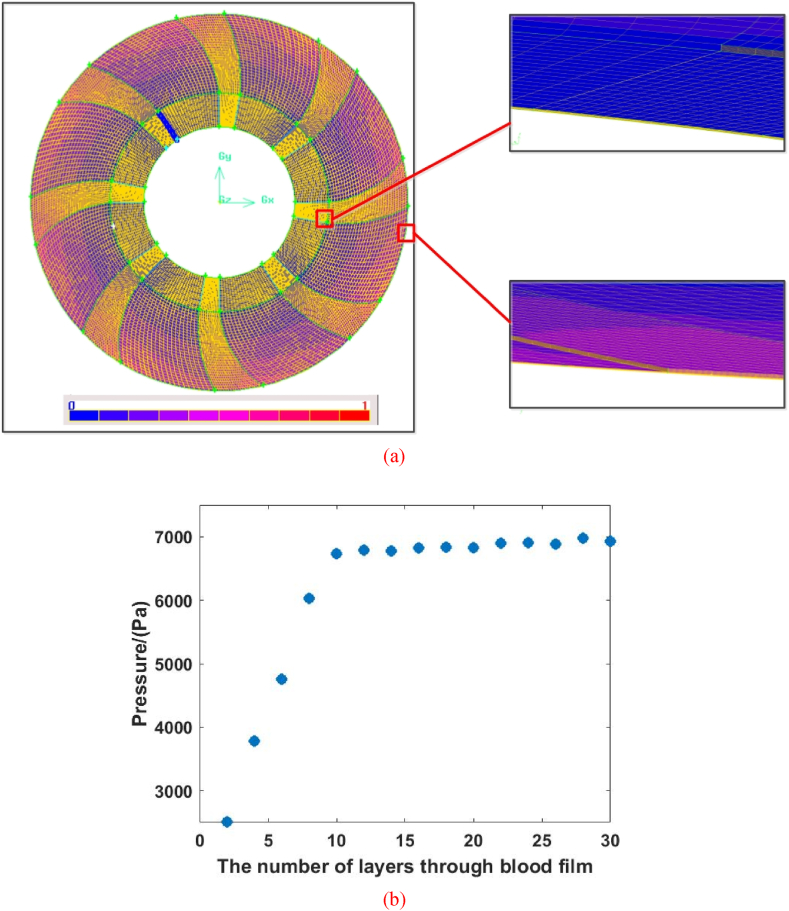
The mesh element and mesh sensitivity of spiral groove bearing: (a) Mesh element; (b) Mesh sensitivity analysis.

In addition, the groove shape plays the key role in the hydrodynamic response of spiral groove bearing. Then, the numerical simulation would be conducted considering the effects of structural parameter variation. The mesh model is shown in Fig. 4 and the simulation parameters are represented in Table 1. Based on the operation condition, the flow rate is defined to 3 L/min at 100 mm Hg pressure. The motion of bearing would cause the high vorticity flow of blood, and the flow state is considered to turbulence. With the best robustness and accuracy, SST k-ω model is employed to represent the hydrodynamic behavior of blood film, which could describe the flow state near the wall with the lower Reynolds number and the characteristics of turbulence in the center region with the higher Reynolds numbers. The model of spiral groove bearing is divided by the hexahedron elements and the relative difference between entrance and export of centrifugal rotary blood pump is considered as 2%. Fig. 4(b) shows that the effects of blood film layer (element number) on the pressure of spiral groove bearing. When the layer is below to 10, the error of pressure is larger. However, the deviation value only 3.49% between 10 layers and 30 layers, and the efficiency of calculation decreases obviously as the layer reaches to 30. And then, the film is divided to 10 layers. The land depth and groove depth are divided 10 layers. According to the equality results, the worst quality of elements is 0.6 and the range of element quality are from 0 (best) to 1 (worst), which is satisfied the requirement of engineering calculation.

**Table 1:** Simulation parameters of spiral groove bearing.

Parameters	value	Parameters	value
Based radius $r_{b}$ (mm)	5	Outer radius $r_{out}$ (mm)	10
Inner radius $r_{in}$ (mm)	5.5, 6, 6.5, 7	Lubricant density ρ (kg/m^3^)	1060
Spiral angle α (°)	20, 25, 30, 35	Lubricant viscosity ν (Pa·s)	0.0036
Groove number n	8, 12, 16, 20	Rotational speed ω (rpm)	3000
Groove width ratio λ	0.5, 0.75, 1, 1.25	Land depth $h_{l}$ (mm)	0.04
Groove depth $h_{g}$ (mm)	0.04, 0.06, 0.08, 0.1	Operation temperature (°C)	36.5

**Fig. 5:**
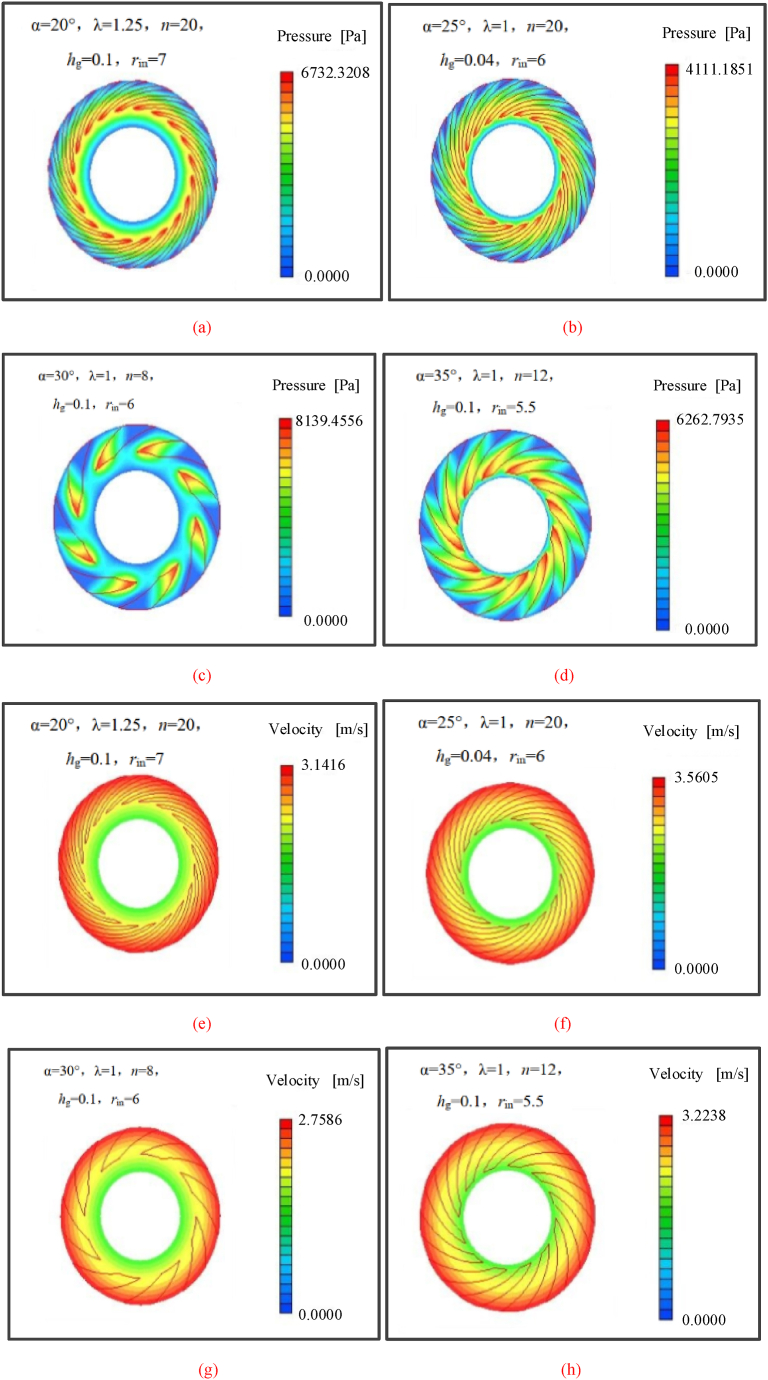
The pressure and velocity distributions of spiral groove bearing with different structural parameters.

The pressure and velocity distribution of spiral groove bearing with different structural parameters are plotted in Fig. 5. It is clear shown that the characteristics of spiral groove could change the pressure distribution and pressure value. In Fig. 5(b), the maximum pressure of blood film is 4111.1851 Pa. As the decrease of groove number (Fig. 5(c)), the maximum pressure of blood film is 8139.4556 Pa. Moreover, the similar phenomenon appears with the change of groove width ratio. The simulation results show that the effects of structural parameters on the hydrodynamic behavior of spiral groove bearing are obvious. The variation of structural parameters would change the flow state of blood film, especially near the center region. It is clear seen that the pressure distribution of blood film for bearing with more groove is more uniform, which could be illustrated that the more groove decreases the variation of velocity gradient for blood and the stability of flow is enhanced. Meanwhile, the velocity distributions of spiral groove bearing are also displayed in Fig. 5(e)–(h). It is can be found that the higher flow velocity of blood film focus on the outer region, and the reason of this phenomenon can be illustrated that the centrifugal force promote the flow velocity of blood film. It is worth noted that the groove feature is closed to the value of flow velocity and the area of lower velocity region is also changed at the same time. Compared with the different structural feature of spiral groove, the maximum value of velocity is opposite with the change of maximum pressure, which is satisfied with the hydrodynamic theory. It is the interesting finding to the investigation of hydrodynamic behavior for spiral groove bearing. Moreover, the load carrying capacity and friction torque could also change at the same time. The simulation results explain that the design of spiral groove has a heavy influence for the performance of spiral groove bearing.

## 4 Results and discussions

In order to reveal the influence of groove shape on the hydrodynamic response of bearing, the spiral angle, groove width, groove number and groove depth are defined as the variables. Then, the numerical simulation can be conducted in detail. Moreover, the orthogonal table is established by the orthogonal design approach and more structural parameters of spiral groove can be considered. The hydrodynamic performance of spiral groove bearing are analyzed by the maximum pressure, load carrying capacity, friction torque and energy loss.

### 4.1 Effects of groove structural parameters

**Fig. 6:**
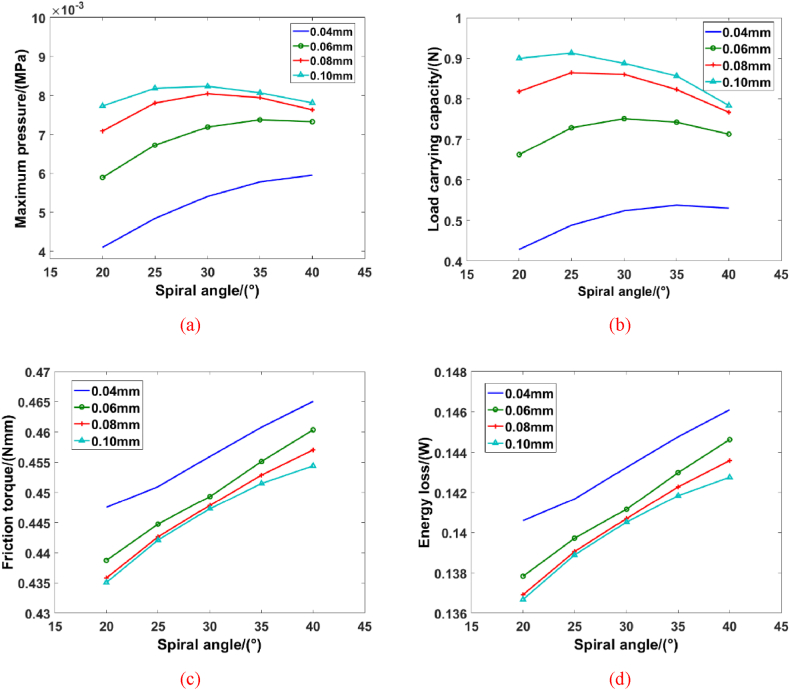
The hydrodynamic performance of spiral groove bearing with different spiral angles: (a) Maximum pressure, (b) Load carrying capacity, (c) Friction torque, (d) Energy loss.

Case 1: In this model, the groove number, groove width ratio and inner radius are defined as 16, 1 and 6 mm, respectively. The values of groove depth are 0.04 mm, 0.06 mm, 0.08 mm and 0.1 mm. The spiral angles are chosen to 20°, 25°, 30°and 35°, respectively. And, the simulation results are shown in Fig. 6. When the groove depth is 0.04 mm, the maximum pressure of blood film increases as the growth of spiral angle, and the load carrying capacity is also changed with the similar phenomenon. The relationship between pressure value and load carrying capacity is close, and the increase of spiral angle could change the velocity gradient of blood flow. And then, the squeeze characteristics of blood molecule is even stronger. As the increase of groove depth, the peak value of maximum pressure appears in the condition (spiral angle is 30°). The characteristics of load carrying capacity is similar with a larger groove depth. However, the load carrying capacity of blood film (*h*_g_ = 0.06 mm) decreases at the spiral angle 35°, as the increases of maximum pressure. Although the maximum pressure increases, the average value of pressure decreases, which can illustrate this phenomenon. Meanwhile, the increase of groove depth would expand the motion region of blood, and the intermolecular force of blood is reduced, which is the reason of decrease of load carrying capacity. As the growth of spiral angle, Fig. 6(c) shows the friction torque and energy loss increases obviously, which means the bigger spiral angle could cause the more energy loss. It is noteworthy that the changed trend of friction torque keep a similar way during the groove depth reaches to 0.08 mm. And, the increase of groove depth could decrease the energy loss. Then, it is seen that the value of spiral angle could decide the gradient of blood flow and flow direction, which is close related to flow force of blood molecule. It is worth noted that the energy loss would change with the growth of friction characteristics.

**Fig. 7:**
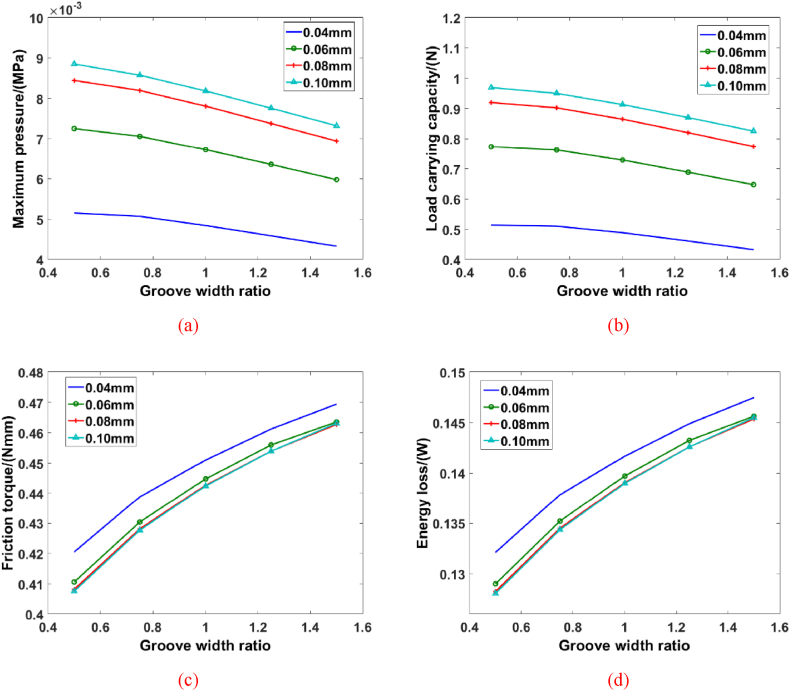
The hydrodynamic performance of spiral groove bearing with different groove width ratios: (a) Maximum pressure, (b) Load carrying capacity, (c) Friction torque, (d) Energy loss.

Case 2: In this model, the groove number is defined as 16, the spiral angel is given as 25° and the inner radius is 6 mm. Fig. 7 displays the mapping relationship between hydrodynamic behavior of spiral groove bearing and groove width ratio. It is clear seen that the growth of groove width ratio could cause the decrease of maximum pressure and load carrying capacity of blood film. When the groove width ratio, the load carrying capacity of blood film is dropping fast especially. Nevertheless, the growth of groove depth could improve the pressure and load carrying capacity of blood film. When the groove width ratio is 0.5 and the groove depth is 0.04 mm, the maximum pressure and load carrying capacity are 5.151 × 10^−3^ MPa and 0.5138 N. With the groove depth expands to 0.1 mm, the maximum pressure and load carrying capacity are 8.851 × 10^−3^ MPa and 0.969 N, respectively. The hydrodynamic characteristics of spiral angle bearing can be explained that the increase of groove width ratio change the blood flow state and the distribution and value of blood-film pressure also change. Moreover, the simulation results shows that the friction torque and energy loss increase as the growth of groove width ratio. Compared with the influence of groove width ratio, the effects of groove depth on the friction torque and energy loss are weak, especially in the condition of a larger groove depth. The increase of groove width ratio could enlarge the contact region area, and the viscosity characteristics of blood would raise the friction force between blood and solid, which would cause the appearance of more energy consumption. As the increase of energy loss, the hydrodynamic performance of blood film would drop and it is not the design objective of spiral groove bearing. In addition, the increase of groove width ratio would reduce the extruded area of blood film, which is the main reason for the decrease of supporting capacity for spiral groove bearing. And then, the increase of clearance between blood molecules would cause the more energy loss during the collision.

**Fig. 8:**
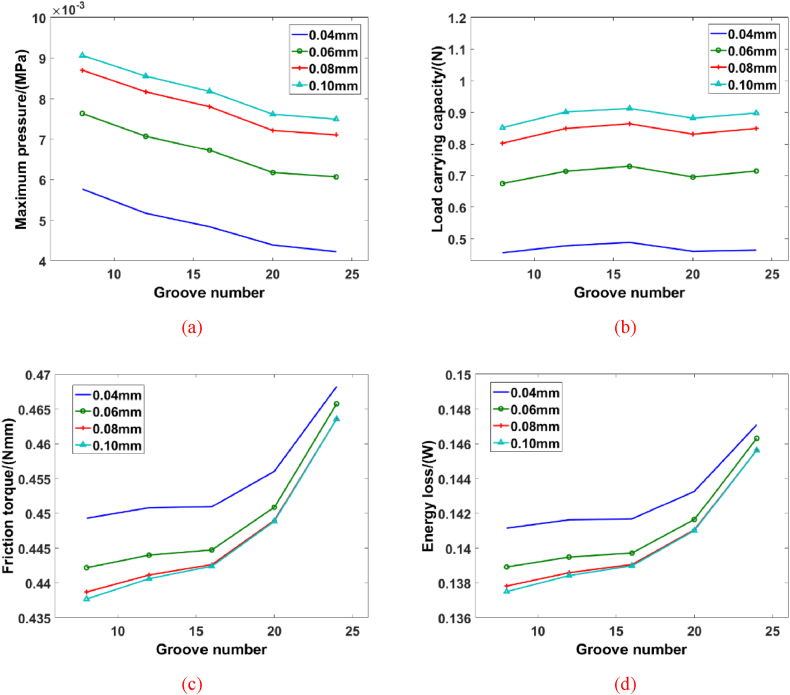
The hydrodynamic performance of spiral groove bearing with different groove numbers: (a) Maximum pressure, (b) Load carrying capacity, (c) Friction torque, (d) Energy loss.

Case 3: The spiral angle, groove width ratio and inner radius are 25°, 1 and 6 mm, respectively. Fig. 8 further represents the hydrodynamic behavior of spiral groove bearing with different structural parameters (groove depth and groove number). It is observed that the maximum pressure of blood film decreases with the growth of groove number. The variation of velocity gradient could change the pressure value and pressure distribution of blood film. According to the simulation results, the variation of groove number changes the load carrying capacity complicatedly. When the groove number is 16, the peak value of load carrying capacity appears. This phenomenon can be concluded that the average value of blood film pressure changes as the increase of groove number, which means that the pressure distribution of blood film also transforms by the variations of groove number. Meanwhile, the friction torque of spiral groove bearing with different structural parameters is presented in Fig. 8(c). When the groove number is 8 and the groove depth is 0.1 mm, the friction torque is 0.4377 Nmm. And, the value of friction torque changes to 0.4424 Nmm, as the groove increases to 16. When the groove number reaches to 20, the friction torque increases to 0.4488 Nmm and the growth rate is 1.45%. This phenomenon illustrates that the more groove could cause the increase of friction force and more energy loss would be produced. Simultaneously, the friction torque of spiral groove bearing almost keeps the same way with the more groove. It is worth concern that the increase of energy loss would decline the load carrying capacity of bearing with more groove number. The more groove number improves the variation frequency of flow state, which promotes the transform between kinetic energy and potential energy of pressure. And then, the separation of boundary layer would appear in the blood film. Then, the rapidly varied flow would cause the appearance of vortex and secondary spiral flow, which is the key point of energy loss. The energy loss is the irreversible physical phenomenon, which could reduce the hydrodynamic ability of spiral groove bearing.

### 4.2 Optimization analysis based on the orthogonal design method

In order to reveal the coupling effects of structural parameters on the hydrodynamic characteristics of spiral bearing, the spiral angle, groove width ratio, groove number, groove depth and inner radius are chosen as the variables. All cases of spiral groove bearing should be conducted at 4^5^ (1024) times, which has a heavy workload. According to the orthogonal design method, the orthogonal table ($L_{16}\left(4^{5}\right)$) can be established and the number of case is 16 times, as shown in Table 2. And, it could also represent the mapping relationship between structural parameters of groove and hydrodynamic behavior of spiral groove bearing. $A_{i}$ denotes the spiral angle (20°, 25°, 30°, 35°), which is correspond to *i* = 1, 2, 3, 4. The groove width ratio and groove number are $B_{i}$ (0.5, 0.75, 1, 1.25) and $C_{i}$ (8, 12, 16, 20). $D_{i}$ and $E_{i}$ represent the groove depth (0.04 mm, 0.06 mm, 0.08 mm, 0.1 mm) and inner spiral radium (5.5 mm, 6 mm, 6.5 mm, 7 mm), respectively. The maximum pressure (MP), load carrying capacity (LCC) and friction torque (FT) are employed as the evaluated indicator. The simulation results are listed in Table 2, which shows that the variation of structural characteristics for spiral groove could change the hydrodynamic characteristics of spiral groove bearing. However, any change of parameters is close to relate with the hydrodynamic response of spiral groove bearing. It is not only related to bearing capacity, but also affects the flow state and intermolecular force. The change of velocity gradient would cause the enlarge of included angle for inter streamline and the energy loss by the tangential stress is also increased. .

**Table 2:** Simulation results.

Case number	$A_{i}$	$B_{i}$	$C_{i}$	$D_{i}$	$E_{i}$	MP (Pa)	LCC (N)	FT (Nmm)
1	1	1	1	1	1	3242.8552	0.330762	0.307309
2	1	2	2	2	2	5683.0679	0.612380	0.321039
3	1	3	3	3	3	6707.7056	0.735465	0.340743
4	1	4	4	4	4	6732.3208	0.746832	0.407694
5	2	1	2	3	4	7939.6304	0.747453	0.374350
6	2	2	1	4	3	8435.6006	0.750174	0.377917
7	2	3	4	1	2	4111.1851	0.440426	0.395081
8	2	4	3	2	1	2921.7131	0.339722	0.398993
9	3	1	3	4	2	7615.9492	0.803692	0.361539
10	3	2	4	3	1	5804.0464	0.523199	0.371483
11	3	3	1	2	4	8139.4556	0.595383	0.405711
12	3	4	2	1	3	5575.3516	0.463980	0.412172
13	4	1	4	2	3	7197.9937	0.718887	0.379957
14	4	2	3	1	4	6137.8115	0.513997	0.404976
15	4	3	2	4	1	6262.7935	0.645613	0.384898
16	4	4	1	3	2	7561.6768	0.625829	0.401533

According to the simulation results, the influence of structural parameters on the hydrodynamic behavior of spiral groove bearing is complicated and confused. Then, the simulation results analysis should be conducted and the optimization design object is also proposed (ODO). The average value ($k_{i}$) and extreme value (*R*) are listed in Table 3. The influence degree of each parameter (IDEP) is obtained by average value and extreme value. The results shows that groove depth ($D_{i}$) plays the important element of hydrodynamic characteristics. The variation of groove depth could relieve the abrupt change of cross-section of blood film, which would obstruct the form conditions of vortex and collision of blood molecules. And then, the decrease of energy loss could provide the helpful for the higher hydrodynamic performance of spiral groove bearing. It is obvious to see that $D_{4}$ and $E_{3}$ are the best values of groove depth and inner radius. The groove width ratio, groove number and spiral angle are $B_{1}$, $C_{2}$ and $A_{4}$, respectively. Then, the structural parameters (D_4_E_3_B_1_C_2_A_4_) could provide a better hydrodynamic performance of spiral groove bearing. Based on the orthogonal design method, the efficiency of structural design for spiral groove bearing in centrifugal rotary blood pump has been improved. However, it should be note that the numbers of parameters and variable value should be satisfied the requirement of orthogonal table, which could represent the coupling relationship of design parameters. More structure parameters and variable values could improve the establishment difficulty of orthogonal table.

**Table 3:** Simulation results analysis.

	$A_{i}$	$B_{i}$	$C_{i}$	$D_{i}$	$E_{i}$	
MP (Pa)	k_1_	5591.4874	6499.1071	6844.8971	4766.8009	4557.8521
	k_2_	5852.0323	6515.1316	6365.2109	5985.5576	6242.9698
	k_3_	6783.7007	6305.2850	5845.7949	7003.2648	6979.1629
	k_4_	6790.0689	5697.7656	5961.3865	7261.6660	7237.3046
	R	1198.5815	817.366	999.1022	2494.8651	2679.4525
	IDEP	E > D > A > C > B				
	ODO	E_4_D_4_A_4_C_4_B_2_				
LCC (N)	k_1_	0.606360	0.650199	0.575537	0.437291	0.459824
	k_2_	0.569444	0.599938	0.617357	0.566593	0.620582
	k_3_	0.596564	0.604222	0.598219	0.657987	0.667127
	k_4_	0.626082	0.544091	0.607336	0.736578	0.650916
	R	0.056638	0.106108	0.041820	0.299287	0.207303
	IDEP	D > E > B > A > C				
	ODO	D_4_E_3_B_1_A_4_C_2_				
FT (Nmm)	k_1_	0.344196	0.355789	0.373118	0.379885	0.365671
	k_2_	0.386585	0.368854	0.373115	0.376425	0.369798
	k_3_	0.387726	0.381608	0.376563	0.372027	0.377697
	k_4_	0.392841	0.405098	0.388554	0.383012	0.398183
	R	0.048645	0.049309	0.015439	0.010985	0.032512
	IDEP	D > C > E > A > B				
	ODO	D_3_C_2_E_3_A_1_B_1_				

## 5 Conclusions

In this paper, the modeling and simulation approach of spiral groove bearing with different structural parameters are proposed based on the CFD technology. The structural characteristics of spiral groove is considered and the viscosity of blood molecules is treated. Firstly, all possible hydrodynamic characteristics of spiral groove bearing are given to the unified description. Then, the mapping relationship between blood film and spiral groove bearing is deduced by the working condition. Secondly, the simulation results are obtained by the presented approach, which is agree quite well with the published data. Then, the three practical examples are considered to illustrate the effects of groove structural parameters on the hydrodynamic behavior of spiral groove bearing. Furthermore, the optimization design of spiral groove bearing is carried out based on orthogonal design method and the optimization design object is proposed. Based on the case study, it is easy to found that more structural parameters of spiral groove are analyzed by the presented method and the case number could be decreased obviously, which improves the computational efficiency of spiral groove bearing.

## Author contribution statement

Cong Xue, Yu Chen: Conceived and designed the experiments; Performed the experiments; Analyzed and interpreted the data; Wrote the paper.

Xiuying Wang: Analyzed and interpreted the data; Contributed reagents, materials, analysis tools or data.

Bin Zhu: Contributed reagents, materials, analysis tools or data.

## Funding statement

Dr Cong Xue was supported by Funding from Young Talent Development Plan of Changzhou Health Commission (2020-233) [No. CZQM2020054].

Xiuying Wang was supported by Changzhou Science and Technology Planning Project [CJ20210067].

## Data availability statement

Data will be made available on request.

## Declaration of interest's statement

The authors declare no conflict of interest.
